# Internal Tremor in Long COVID May Be a Symptom of Dysautonomia and Small Fiber Neuropathy

**DOI:** 10.3390/neurolint17010002

**Published:** 2024-12-25

**Authors:** Svetlana Blitshteyn, Ilene S. Ruhoy, Lauren R. Natbony, David S. Saperstein

**Affiliations:** 1 Department of Neurology, School of Medicine and Biomedical Sciences, University at Buffalo Jacobs, Buffalo, NY 14203, USA; 2 Dysautonomia Clinic, Williamsville, NY 14221, USA; 3 Department of Neurology, Mount Sinai South Nassau, Oceanside, NY 11572, USA; ileneruhoymdphd@gmail.com; 4 Department of Neurology, Icahn School of Medicine at Mount Sinai, New York, NY 10029, USA; 5 Integrative Headache Medicine of New York, New York, NY 10016, USA; 6 Center for Complex Neurology, University of Arizona College of Medicine, Phoenix, AZ 85004, USA

**Keywords:** internal tremor, Long COVID, POTS, small fiber neuropathy, intravenous saline

## Abstract

**Background/Objectives:** Internal tremor (IT) is often reported by patients with post-acute sequelae of SARS-CoV-2, also known as Long COVID, as a distressing and disabling symptom. Similarly, physicians are typically perplexed by the nature and etiology of IT and find it extremely challenging to manage. **Methods:** We describe a patient with Long COVID who experienced IT as part of post-COVID postural orthostatic tachycardia syndrome (POTS) and small fiber neuropathy (SFN) and review the limited literature available on this topic. **Results:** Our patient’s IT improved significantly after intravenous saline infusions, but there was no effect on IT with oral hydration, increased oral sodium chloride intake, neuropathic pain medications, muscle relaxants, or medications used for the treatment of POTS. **Conclusions:** Based on this case, our clinical experience, and the limited literature available to date, we believe IT is a manifestation of POTS and SFN, which may be driven by hypovolemia, cerebral hypoperfusion, sympathetic overactivity, neuropathic pain, and mast cell hyperactivation. Subjective description, objective findings, and diagnostic and therapeutic considerations in patients with IT and Long COVID are discussed.

## 1. Introduction

Internal tremor (IT), also referred to as internal vibrations, has been reported as one of many numerous, diverse, and multisystemic symptoms of Long COVID and was found to occur in 37% of patients in a cross-sectional study of 423 people with Long COVID [1,2]. Importantly, it was demonstrated in one study that, compared to people with Long COVID who did not experience IT, people with Long COVID and IT had higher rates of new-onset mast cell disorders (11% vs. 2.6%); neurologic conditions, including small fiber neuropathy (SFN) (22% vs. 8.3%); postural orthostatic tachycardia syndrome (POTS) (33% vs. 11%); and myalgic encephalomyelitis/chronic fatigue syndrome (ME/CFS) (21% vs. 11%) [1].

Scientific literature on IT is extremely limited, but it has been known to occur in clinical practice in patients with POTS, mast cell activation syndrome (MCAS), and other neurologic conditions involving a hyperadrenergic state. IT is often described by patients as a feeling or a sensation that they experience internally and that is not visible to the examiner, making it a symptom rather than a sign, unlike tremors observed in movement disorders, such as benign essential tremor or Parkinson’s disease. Here, we present a case of a patient with IT and post-COVID POTS and SFN who experienced significant improvement of IT after receiving intravenous saline, suggesting that IT may be a symptom of POTS and SFN.

## 2. Case Presentation

A woman in her mid-forties with a history of episodic migraine and seasonal allergies developed rapid heart rate, dizziness, body pain, fatigue, exercise intolerance, and brain fog after experiencing a mild SARS-CoV-2 infection. She also reported having IT that was not visible to others, which she described as vibration, trembling, and internal shaking that predominantly involved her legs and torso. All symptoms persisted for the following 10 months, resulting in significant functional impairment and inability to work at her previous job. The patient had no pre-existing personal or family history of the autoimmune/inflammatory, post-infectious, or genetic disorders. A physical exam was remarkable for purple–blue discoloration of the feet when the patient was sitting down, which was consistent with acrocyanosis. A musculoskeletal exam showed mild hypermobility, with the patient scoring 5/9 on a Beighton scale. Cardiac and pulmonary exams were unremarkable. A neurologic exam was only remarkable for mild loss of temperature sensation in the feet, but other sensations were intact. No visible tremor, myoclonus, muscle weakness or abnormal reflexes were present on neurologic examination. The patient had no evidence of rigidity, spasticity, bradykinesia, ataxia, or abnormal gait. There were no signs of functional neurologic disorder on physical examination.

Diagnostic investigations in this patient were conducted according to the consensus guidance statement on the assessment and treatment of autonomic dysfunction in patients with post-acute sequelae of SARS-CoV-2 infection [3]. A 10 min stand test performed at an outpatient clinic revealed a supine heart rate of 75 bpm with a blood pressure of 110/70 and a standing heart rate of 118 bpm with a blood pressure of 100/74 after 10 min of standing accompanied by dizziness, consistent with the diagnostic criteria for POTS [4]. A skin biopsy showed reduced epidermal nerve fiber density at the foot and normal epidermal nerve fiber density at the thigh; sweat gland density was significantly reduced at both sites. Based on the patient’s symptoms and a 10 min stand test, the patient was diagnosed with POTS and SFN, confirmed with a skin biopsy. MRI of the brain, 2D cardiac echocardiography, chest X-ray and electrocardiogram were all unremarkable. A 24 h Holter monitor demonstrated an average heart rate of 80 beats/min and a maximum heart rate of 140 beats/min, without evidence of supraventricular tachycardia or other types of arrhythmia.

Blood tests revealed no abnormalities, including complete blood cell count, comprehensive metabolic panel, magnesium, and thyroid function tests. Hemoglobin, renal function, liver function, serum sodium, and blood glucose levels were within the normal limits, without signs of anemia, acute renal injury, or liver dysfunction. The 24 h urine catecholamines were within the normal limits. Serum vitamin B12, vitamin B1, vitamin B6, and vitamin D25-OH levels were normal; ANA was elevated at 1:160 with a homogenous pattern, but anti-DS DNA, anti-SSA, and anti-SSB antibodies; anti-thyroid antibodies; anti-CCP antibodies; anti-ganglionic acetylcholine receptor antibodies; ESR; CRP; and RF were within the normal limits.

Differential diagnoses included post-COVID cardiac arrhythmia, cardiomyopathy, and myocarditis, which were ruled out via unremarkable cardiovascular tests. Additionally, anemia, electrolyte abnormalities, thyroid disease, pheochromocytoma, Addison’s disease, vitamin deficiencies, and defined autoimmune disorders, including paraneoplastic syndromes, were ruled out via blood tests and a lack of clinical features of lupus, Sjogren’s syndrome, rheumatoid arthritis, and malignancy. However, an autoimmune basis of post-COVID POTS was evident given a positive ANA, comorbid SFN, and post-infectious onset of symptomatology. It was previously demonstrated pre-COVID-19 pandemic that patients with POTS have a higher prevalence of positive ANA and other autoimmune markers than the general population [5].

Treatment with non-pharmacologic and pharmacologic therapies was instituted according to the consensus guidance statement on the assessment and treatment of autonomic dysfunction in patients with post-acute sequelae of SARS-CoV-2 infection [3]. Non-pharmacologic therapies with increased oral fluids and salt intake, waist-high compression stockings, and recumbent exercise were implemented, but the patient experienced no significant improvement with these treatment modalities. She was subsequently started on medications for POTS, such as fludrocortisone and atenolol, and her autonomic symptoms, including dizziness, palpitations, and exercise intolerance, improved. However, IT persisted and became one of her most bothersome symptoms. When she experienced an exacerbation of POTS symptoms, she received 1 L of intravenous saline in the emergency department, which resulted in significant improvement of IT. Neither oral fluids and salt nor the medications tried for neuropathic pain previously, such as gabapentin, pregabalin, duloxetine, Flexeril, Zanaflex, and low-dose naltrexone, were as effective as intravenous saline, according to the patient.

With the shared decision making, the patient elected to continue receiving intermittent intravenous saline infusions every 2–3 weeks through a peripheral venous access. Her symptoms of POTS and IT were improved with intravenous hydration in a way that was not possible to achieve with oral hydration and increased oral sodium chloride intake. However, she continued to have POTS and was unable to return to work.

## 3. Discussion

Unlike the tremor of movement disorders, such as benign essential tremor and Parkinson’s disease, which is visible to the examiner, IT typically cannot be seen externally and, therefore, cannot be assessed or quantified objectively on a neurologic exam (Table 1). Patients commonly describe IT as a feeling of vibration, twitching, tremoring, shaking, trembling, or other adjectives that affects a part of the body or the entire body [2]. Per the patients’ descriptions, IT usually varies in intensity depending on the time of day and severity of other Long COVID symptoms, typically being worse in the morning and when other symptoms of Long COVID, such as fatigue, brain fog, dizziness, and sleep disturbance, are exacerbated.

In our experience, various treatment modalities for IT can be utilized, consisting of neuropathic pain medications, such as gabapentin, pregabalin, or duloxetine; muscle relaxants, such as Flexeril or Zanaflex; and dysautonomia treatment options, such as beta blockers, pyridostigmine, and clonidine [3]. Here, we present an illustrative case of a patient with IT and confirmed diagnoses of POTS and small fiber neuropathy that began after SARS-CoV-2 infection. She experienced temporary cessation of IT after each intravenous saline infusion. We have other patients with POTS, SFN, hypermobility spectrum disorders, and mast cell activation syndrome, whether they developed these syndromes after SARS-CoV-2 infection or before COVID-19 pandemic, who similarly experienced temporary improvement or cessation of IT after treatment with intravenous saline. Based on these reports and our collective experience treating patients with POTS and SFN before and after the COVID-19 pandemic, we believe that IT represents a feature of dysautonomia and small fiber neuropathy that may be caused by hypovolemia, cerebral hypoperfusion, hyperadrenergic state, mast cell hyperactivation, and neuropathic processes affecting the small nerve fibers and other possible mechanisms of dysautonomia that have yet to be elucidated (Table 1). Importantly, the findings from the one and only study available to date on IT in 423 people with Long COVID confirm our clinical experience by describing higher rates of MCAS, SFN, and POTS in patients with IT compared to those without IT [1].

In clinical practice, IT is often mis-attributed to anxiety, depression, or functional neurologic disorder because self-reported tremors, vibrations, and shaking are not corroborated by physical exam findings, which typically show no objective evidence of tremor, although, in some cases, mild postural tremor may be observed. In our clinical experience, therapeutic modalities for anxiety, depression, or functional neurologic disorder have been largely ineffective in reducing or eliminating IT in patients with POTS and SFN. Similarly, based on our observation, traditional therapies for neuropathic pain, such as anticonvulsants, anticholinergics, SSRIs, or SNRIs, have not been particularly effective in diminishing the unpleasant sensations of IT. Anecdotally, we found that treatment of POTS with therapies targeting sympathetic overactivity, hypovolemia, and mast cell hyperactivity has been more efficacious than neuropathic pain medications. These therapies consist of beta blockers; increased oral fluids and salt intake; intravenous fluids; and other medications, such as pyridostigmine, which enhances parasympathetic transmission; midodrine, which increases vasoconstriction and therefore improves venous return and cerebral perfusion; and benzodiazepines, which may reduce IT via GABA-mediated actions in the central autonomic networks (Table 1). Obviously, chronic benzodiazepine use is not routinely recommended due to its addictive potential and should be used either temporarily or as a last resort in treatment-refractory, severe cases of IT.

POTS and SFN are comorbid conditions, with both being highly prevalent in patients with Long COVID: an estimated 30–75% of patients with Long COVID have POTS [6,7], and nearly 70% have autonomic dysfunction [8]. The prevalence of SFN in Long COVID is unknown, but appears to be similarly high and close to 70% based on small studies [9,10]. Additionally, mast cell hyperactivity has been also reported in patients with Long COVID [11]. Similar to Long COVID patients, the spectrum of symptomatology in patients with POTS and SFN is broad and usually includes dizziness, palpitations, fatigue, exercise intolerance, cognitive disturbance, and headache. Diagnostic testing for patients with POTS and SFN includes a 10 min stand test or a tilt table test, a skin biopsy to assess for epidermal nerve fiber density and sweat gland density, and/or a quantitative sudomotor axon reflex test as part of the autonomic function tests, if available [3].

Why intravenous fluids appear to be more effective than oral fluid and salt intake in patients with POTS has not been fully elucidated, but intravenous saline has long been known to be beneficial in patients with severe POTS [12,13,14]. However, reliance on intravenous fluids as a form of chronic therapy is typically not recommended, mainly due to the risk of sepsis and thrombosis at the site of the catheter if administered through a central venous line or a port [3]. Nonetheless, the use of intravenous saline every 2–3 weeks through the peripheral vein has not been formally investigated in patients with POTS or Long COVID, some of whom have been using intermittent IV saline therapy for years with anecdotally reported good outcomes. It is likely that the same mechanisms that underlie the intravenous fluid’s effectiveness in temporarily reducing the symptoms of POTS may also be involved in temporarily decreasing or eliminating IT in patients with Long COVID [12,13,14,15]. As this is a case report of one patient, further studies, including large case series and placebo-controlled studies, are necessary to understand the mechanisms of IT and the benefits of intravenous saline, as well as to determine whether intravenous saline every 2–4 weeks via peripheral venous access may be utilized as a safe and effective chronic therapy in patients with POTS and Long COVID, including those with IT. Additionally, as self-reports and lived experiences of IT in patients with Long COVID are numerous and the scientific literature on IT is extremely limited [1,2], clinical evaluation and future studies should focus on assessing patients for autonomic dysfunction and SFN to avoid misdiagnosis and provide appropriate treatment.

## 4. Patient Perspective

I developed internal tremor as part of Long COVID after a mild COVID-19 infection. The internal tremor feels as though someone is running an electrical current through my body continuously. The current, or vibrations, are most pronounced in the upper torso but when it’s even more intense it feels as though my thighs are also subjected to the same current or vibration. They take up all my energy and can last all day as they did for the first several months of my Long COVID, and they left me unable to do anything including walk, dress myself, or brush my teeth. Subsequently, I started IV saline therapy, first weekly and now twice a month. For the first few months just getting the IV therapy was taxing, and it took a few days for improvement of my symptoms. Although the vibrations did not fully abate, they were far less intense, allowing me a small amount of energy. Prior to this, there was no symptom improvement with any other therapies. Now, when I get an IV, I have some internal tremor during the IV and maybe for an hour afterwards but then the tremor actually disappears for several days and only returns when I have taxed myself by trying to move around too much. As I get closer to my next two-week IV date, the tremor will return for longer periods of time and become more frequent, but it’s still not as bad as it was during the first eight months of my Long COVID.

## 5. Conclusions

Based on the case presented here, our collective clinical experience, and the limited literature available to date on IT, we believe IT is a manifestation of POTS and SFN, which may be driven by hypovolemia, cerebral hypoperfusion, sympathetic overactivity, neuropathic pain, and mast cell hyperactivation. We emphasize the following learning points, which are essential to the care of patients with Long COVID:

- Clinicians should consider evaluation for postural orthostatic tachycardia syndrome and small fiber neuropathy in patients who report internal tremor (IT) after SARS-CoV-2 infection.
- Misdiagnosis with anxiety or functional neurologic disorder is common because, unlike tremors of movement disorders, IT is usually not visible to the examiner.
- Treatment of IT with therapies targeted to improve hypovolemia, sympathetic overactivity, cerebral hypoperfusion, and neuropathic pain can be trialed, including intermittent intravenous saline administered through a peripheral venous line.

## Figures and Tables

**Table 1:** Clinical features of internal tremor in Long COVID, possible associated diagnoses, and therapeutic considerations.

Subjective Description	Objective Findings	Possible Associated Diagnoses	Possible Therapies
Internal vibrations	Usually no evidence of tremor	POTS	Beta blockers (propranolol, atenolol)
Internal shaking	Mild postural tremor may be present	Orthostatic hypotension	Midodrine
Internal trembling	May have decreased temperature and pain sensation distally or patchy	Neurocardiogenic syncope	Fludrocortisone
Internal buzzing	Usually no rigidity, bradykinesia, or muscle weakness or wasting	Autonomic dysfunction if 10 min stand test or a tilt table test is unremarkable	Pyridostigmine
Internal twitching	May have abnormal 10 min stand test	Small fiber neuropathy	Clonidine or guanfacine
Internal swaying	May have purplish discoloration of the feet and/or hands	Large fiber sensory neuropathy	Intravenous saline
Internal movement	May have joint hypermobility	Chronic fatigue syndrome	Low-dose naltrexone
Internal motion	May have dermographism	Fibromyalgia	Hydroxyzine
Internal jolts	May have flushing	Hypovolemia and/or cerebral hypoperfusion	Clonazepam or diazepam (short-term use for severe cases only)

## Data Availability

Data is contained within the article.
